# Long-term seizure outcome with the surgically remediable syndrome of frontal lobe epilepsy associated with superior frontal sulcus-related dysplasia

**DOI:** 10.3389/fneur.2023.1096712

**Published:** 2023-03-23

**Authors:** Yan Xu, Wen-Han Hu, Xiao-Qiu Shao, Yan-Shan Ma, Lin Lou, Kai Zhang, Jian-Guo Zhang

**Affiliations:** ^1^Department of Neurosurgery, Beijing Tiantan Hospital, Capital Medical University, Beijing, China; ^2^Department of Functional and Stereotactic Neurosurgery, Beijing Neurosurgical Institute, Capital Medical University, Beijing, China; ^3^Department of Neurosurgery, Epilepsy Center, Zhejiang Provincial People's Hospital (Affiliated People's Hospital, Hangzhou Medical College), Hangzhou, Zhejiang, China; ^4^Department of Epilepsy, Beijing Tiantan Hospital, Capital Medical University, Beijing, China; ^5^Department of Neurosurgery, Beijing Fengtai Hospital, Beijing, China

**Keywords:** frontal lobe epilepsy (FLE), superior frontal sulcus (SFS) related dysplasia, focal cortical dysplasia (FCD), epilepsy surgery, long-term outcome assessment

## Abstract

**Objective:**

To assess the long-term outcome of the surgically remediable syndrome of frontal lobe epilepsy (FLE) associated with superior frontal sulcus (SFS)-related dysplasia.

**Methods:**

We retrospectively reviewed the medical charts and surgical features of 31 patients with drug-resistant frontal lobe epilepsy in our centers between 2016 and 2018. All patients underwent surgical resection. According to the epileptogenic zone (EZ), localization and resection extent were classified as (1) pure SFS group (PS group), (2) associated SFS group (AS group), and (3) no SFS group (NS group). The general characteristics, neuroradiological findings, morbidity, pathology, and long-term seizure outcome after surgery were analyzed to extract the potential value of the surgery for SFS-related dysplasia.

**Results:**

Of 31 patients with FLE who underwent epilepsy surgery, 15 patients (nine men) were included PS group, five patients (five men) in the AS group, and 11 patients (eight men) in the NS group. Eleven patients detected abnormal focal signals in the presurgical MRI. Six patients in the PS group demonstrated the suspected focal cortical dysplasia (FCD) in the SFS detected with MRI. All patients demonstrated focal abnormal hypometabolism foci in the PET-MR co-registration. Twenty-five patients (80.6%) were seizure-free since surgery, including all 15 patients (100%) of the PS group, three in five patients (60%) of the AS group, and seven in 11 patients (63.6%) of the NS group. The difference in outcome between different groups was significant (*p* = 0.004, PS vs. AS group; *p* = 0.005, PS vs. NS group). As of the last follow-up (mean 66.2 ± 9.7months), 25 patients (80.6%) were seizure-free since surgery (Engel's class I). In addition, antiseizure medication was withdrawn in 19 patients (61.3%). Histologic examination of resected specimens revealed FCD in all 31 patients. The percentage of FCD II type was 100, 60, and 63.6% in the three different groups, respectively.

**Conclusion:**

SFS-related dysplasia is a neuropathologic entity with a favorable postoperative outcome. FCD II is the most common type of SFS-related dysplasia. FDG-PET co-registered with MRI should be performed in patients with suspected SFS-related dysplasia, since it may depict areas of hypometabolism suggestive of dysplasia in the absence of MRI abnormalities.

## 1. Introduction

Frontal lobe epilepsy (FLE) is the most refractory extratemporal focal epilepsy benefitting from surgical treatment. The long-term seizure freedom rate after surgery is 13–47% (1–3). Although MR-positive FLE patients have a higher seizure freedom rate after epilepsy surgery than those regarded as MR-negative (4), the topography relationship between the MRI lesion and the epileptogenic zone (EZ) is still complex, and the EZ is often not restricted to the cortex immediately surrounding the MRI lesion, thus making the surgical decision more challenging. Certainly, it is much more difficult to identify the EZ in MR-negative FLE (5, 6). Therefore, the identification of subtle morphological markers or regular dysplasia locations through the standard neuroradiological images and postprocessing, and inter/ictal electrophysiology, is particularly important in this difficult-to-treat group of patients.

It is difficult to make precise morphological analysis and EZ identification before surgery because of the substantial variable and complex characteristics of the cerebral cortex surface. However, small focal cortical dysplasia (not) detected with MRI is often found on pathological examination, particularly in the depth of the posterior part of the superior frontal sulcus (SFS) (7, 8). The MRI features of dysplasia in the superior frontal sulcus depth are cortical thickening, gray-white junction blurring, and subcortical T2/flair hyperintensity, sometimes pointing to the ventricle as a “transmantle sign” (9–12). Furthermore, it is always possible to find the dysplastic sulcus straightening and elongation, and the overlying cerebral surface depression in the visible MRI image (11, 13, 14).

This study aimed to identify neuroradiological findings, morbidity, pathology, and long-term seizure outcome features that might aid the detection of patients with SFS-related dysplasia among drug-resistant FLE. We attempt to evaluate the existence of SFS-related dysplasia patterns and their relationship to the EZ in FLE surgery patients.

## 2. Methods

### 2.1. Patient selection

We retrospectively reviewed the data of 31 consecutive patients (22 men), selected from the epilepsy center database of Beijing Tiantan Hospital (24 patients) and Zhejiang Provincial People's Hospital (seven patients), who, after selection, (1) underwent presurgical evaluation according to the same protocol, (2) underwent frontal lobe epilepsy surgery between 2016 and 2018 in our epilepsy centers, and (3) were followed up >36 months (range 43–75 months). Patients and their supervisors gave informed written consent.

The presurgical evaluation included gathering a comprehensive medical history, neurological examination, neuropsychological assessment, long-term scalp video-EEG (including interictal/ictal EEG and semiology), 3T high-resolution MRI, and interictal positron emission tomography (PET) scan in all patients. Preoperative data were reviewed in a multidisciplinary meeting of the epilepsy center before proceeding with invasive procedures or immediate surgery.

### 2.2. Pre- and postsurgical MRI findings, and presurgical interictal PET protocol

The presurgical and all consecutive postsurgical 3T MRI scans of all 31 patients, including 3D T1 MPRAGE (1^*^1^*^1), axial T1 (3 mm thickness with 0 mm gap), T2, and fluid-attenuated inversion recovery (Flair) (axial and coronal 3 mm thickness with 0 mm gap), were reviewed by two experienced neuroradiologists. PET scans were acquired in the interictal state under standard resting conditions using SIEMENS Biograph64_mCT PET/CT scanners. Approximately 45 min following the intravenous administration of 2.6–13.2 mCi 18F-labeled fluoro-2-deoxy-D-glucose (FDG), 3D PET images of the brain were obtained from the vertex to the skull base (slice thickness 3 mm). Images were attenuation-corrected using non-contrast CT transmission information. Interictal PET and 3D T1 were routinely co-registered for all 31 patients with the software SPM12 (UCL, www.fil.ion.UCL.uk/spm). According to the presurgical radiological features of the lesions, we classified two groups as (1) MR-positive and MR-negative, and (2) PET-positive and PET-negative. The EZ localization and resection extent were classified as (1) pure SFS group (PS group), which included ictal semiology and scalp EEG consistent with a corresponding well-delineated lesion in SFS—the lesion was considered the EZ, and the resection was focal lesion oriented; (2) associated SFS group (AS group), which included not only SFS-related dysplasia but also the neighbored cortex in frontal lobe; and (3) no SFS-related group (NS group), which excluded SFS-related EZ in frontal lobe.

We defined the patient as MR-negative when standard quality structural cerebral MRI was considered normal or non-localizing by the neuroradiologist and the epileptologist at the time of the decision to perform invasive recordings or surgery.

### 2.3. Pre- and postsurgical EEG findings

In presurgical scalp EEG, interictal epileptic discharges and ictal onset patterns were classified as (1) focal, involving only the lesion-related areas and neighbor cortex, or (2) non-focal. Data from MRI, interictal PET, PET-MR co-registration, ictal semiology, and interictal/ictal scalp EEG were inconclusive or inconsistent in order to define an area of resection in 21 patients who then underwent intracranial EEG recording using the SEEG approach. A stereotactic device or robotic arm guided the implantation of SEEG throughout all the non-invasive examinations.

### 2.4. Surgical procedures and histopathology

All patients underwent surgery based on the decision made by the multidisciplinary groups of the epilepsy center, according to the culmination of clinical semiology, neuro-radiological and post-processing images (PET-MR co-registration), and electrical investigation data leading to the location and extent of the EZ. Focal cortical resection, or corticectomy, was performed, with the type and extent of corticectomy determined by the size and location of the EZ. Resection surgery removes the entire epileptogenic lesion and the associated EZ, as defined by MRI visible lesions and long-term invasive EEG recordings. In cases in the PS group with ictal semiology and scalp EEG consistent with a corresponding well-delineated lesion in SFS, the lesion was considered the EZ, and the resection was focal lesion-oriented. In cases with (1) electroclinical data inconsistent with the corresponding lesion, (2) a lesion located into or closed to the eloquent cortex, or (3) a blurred-margin lesion or MRI invisible lesion, the EZ was defined by (1) MRI analysis and verified by co-registration with interictal PET, and (2) long-term SEEG recording and intracranial stimulation studies after chronic SEEG recording to identify eloquent cortex (frequency: 50 Hz, current: 1 mA-10 mA, pulse width: 200 μs). All surgical specimens were ascertained by histopathology.

### 2.5. Seizure outcomes

The postoperative outcome was usually accessed at 1, 3, and 6 months, and then once a year. Possible neurologic deficits were noticed, and the rate of seizure frequency reduction was assessed with respect to the preoperative period according to Engel's classification system. Postoperative withdrawal of antiseizure medication commenced between 2 and 5 years after successful epilepsy surgery.

### 2.6. Statistical analysis

Fisher's exact test was used to test for the duration of seizure and outcome in different groups. Statistical tests were performed with the software Statistical Package for the Social Sciences (SPSS) for Windows, version 26. *P*-values of <0.05 were considered statistically significant.

## 3. Results

### 3.1. Patient characteristics

**Figure 1:**
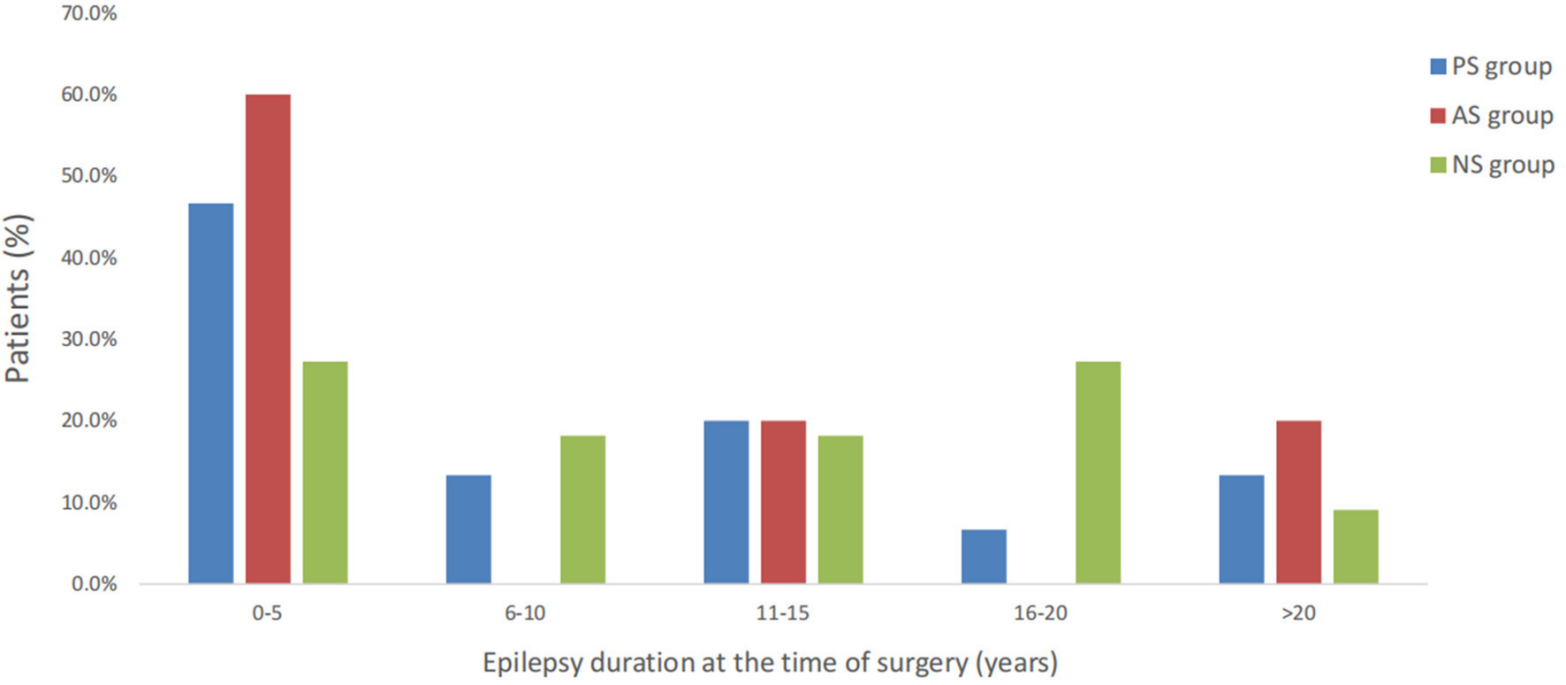
Distribution of epilepsy duration at the time of surgery was similar during three different groups.

Of 31 patients with FLE who underwent epilepsy surgery, most were men: 15 patients (nine males) were included PS group (Table 1), five patients (five males) were included AS group (Table 2), and 11 patients (eight males) were included in NS group (Table 3). The mean age was 15.7 years (range 4–36 years), 15.2 years (range 7–27 years), and 20.0 years (range 3–37 years), for each of the groups, respectively. The mean epilepsy duration at the time of surgery was 9.5, 7.4, and 10.7 years, for each of the groups, respectively. The distribution of the epilepsy duration was similar, and there was no significant difference in the epilepsy duration at the time of surgery in these three groups (Figure 1). Fourteen patients (5/15 in the PS group, 1/5 in AS group, and 8/11 in the NS group) had seizure attacks predominantly at night or during sleep, and the difference was significant (*p* = 0.011) between the SFS-related groups (PS group and AS group) and other FLE (NS group). Two patients (one in the PS group and one in the NS group) had seizures during the daytime. All patients had refractory epilepsy despite appropriate antiseizure medication.

**Table 1:** Demographic features and clinical manifestations of the PS group.

**Patient**	**Gender**	**Age (years)**	**Duration of epilepsy(years)**	**Epileptic syndrome**	**Frequency**	**3T-MRI with FLAIR**	**PET-MRI**	**SEEG**	**Surgical complications**	**Pathology**	**Engel class**	**Follow up (months)**
1	M	25	17	Focal RF	Nightly	N	P	Yes	N	FCD IIa	I	69
2	F	19	10	Focal LF	Weekly	N	P	Yes	N	FCD IIa	I	71
3	M	28	13	Focal LF	Daily	N	P	Yes	Transient mild Rt motor deficits	FCD IIa	I	73
4	M	4	4	Focal LF	Daily	Lt posterior SFS	P	No	Transient mild Rt motor deficits	FCD IIb	I	67
5	F	3	3	Focal LF	Daily	Lt posterior SFS	P	No	Transient mild Rt motor deficits	FCD IIb	I	70
6	M	17	12	Focal RF	Monthly	Rt posterior SFS	P	No	N	FCD IIa	I	75
7	F	4	2	Focal LF	Monthly	N	P	No	N	FCD IIa	I	74
8	F	22	13	Focal RF	Daily	N	P	No	Transient mild Lt motor deficits	FCD IIb	I	74
9	M	24	21	Focal LF	Weekly	N	P	Yes	N	FCD IIa	I	72
10	M	36	27	Focal LF	Daily	Rt anterior SFS	P	Yes	N	FCD IIb	I	60
11	M	6	3	Focal LF	Daily	N	P	Yes	N	FCD II a	I	59
12	M	6	3	Focal LF	Daily	Lt middle and posterior SFS	P	Yes	N	FCD II a	I	47
13	F	5	4	Focal LF	Daily	Lt posterior SFS	P	No	Transient mild Rt motor deficits	FCD IIb	I	43
14	M	12	4	Focal RF	Daily	N	P	Yes	Transient mild Lt motor deficits	FCD II a	I	43
15	F	24	7	Focal RF	Daily	N	P	Yes	Transient mild Lt motor deficits	FCD II a	I	66

M, male; F, female; LF, left frontal; RF, right frontal; Lt, left; Rt, right; N, negative; P, positive; FCD, focal cortical dysplasia.

**Table 2:** Demographic features and clinical manifestations of the AS group.

**Patient**	**Gender**	**Age (years)**	**Duration of epilepsy (years)**	**Epileptic syndrome**	**Frequency**	**3T-MRI with FLAIR**	**PET-MRI**	**SEEG**	**Surgical complications**	**Pathology**	**Engel class**	**Follow up(months)**
1	M	8	1	Focal RF	Nightly	N	P	N	N	FCD I	I	74
2	M	27	21	Focal LF	Daily	N	P	Y	N	FCD IIa	II	72
3	M	12	1	Focal LF	Daily	N	P	Y	Transient mild Rt motor deficits	FCD II b	I	68
4	M	7	1	focal RF	Daily	Rt anterior part of superior frontal lobe and anterior SFS	P	Y	N	FCD II b	I	62
5	M	22	13	Focal LF	Daily	Lt precentral, premotor and posterior SFS	P	Y	Transient mild Rt motor deficits	FCD	III	50

**Table 3:** Demographic features and clinical manifestations of the NS group.

**Patient**	**Gender**	**Age (years)**	**Duration of epilepsy (years)**	**Epileptic syndrome**	**Frequency**	**3T-MRI with FLAIR**	**PET-MRI**	**SEEG**	**Surgical Complications**	**Pathology**	**Engel class**	**Follow up(months)**
1	M	16	2	Focal RF	Monthly	N	P	Y	N	FCD IIa	I	70
2	F	17	11	Focal RF	Monthly	N	P	Y	N	FCD IIa	I	75
3	M	28	26	Local RF and/or insular	Daily	N	P	N	N	FCD I	I	75
4	M	25	16	Focal LF	Daily	Lt premotor	P	N	Transient mild Rt motor deficits	FCD IIb	I	74
5	M	3	2	Local OFC, ACC and/or insular	Daily	LT ACC and rectus	P	Y	N	FCD IIb	II	74
6	M	20	16	Local OFC and/or ACC	Daily	N	P	Y	N	FCD II a	I	73
7	M	21	7	Focal RF	Weekly	N	P	Y	N	FCD I a	II	69
8	F	22	17	Local frontal base, insular, ACC, and/or amygdala	Daily	N	P	Y	N	FCD II a	I	73
9	F	13	12	Local frontal base, insular, ACC, and/or amygdala	Daily	N	P	Y	N	FCD I	III	58
10	M	18	6	Focal LF	Daily	Lt anterior inferior frontal sulcus	P	N	N	FCD II b	I	67
11	M	37	3	Focal LF	Daily	N	P	Y	N	FCD I	II	54

OFC, orbitofrontal cortex; ACC, anterior cingulate cortex.

### 3.2. Presurgical MRI, PET findings, and SEEG

**Figure 2:**
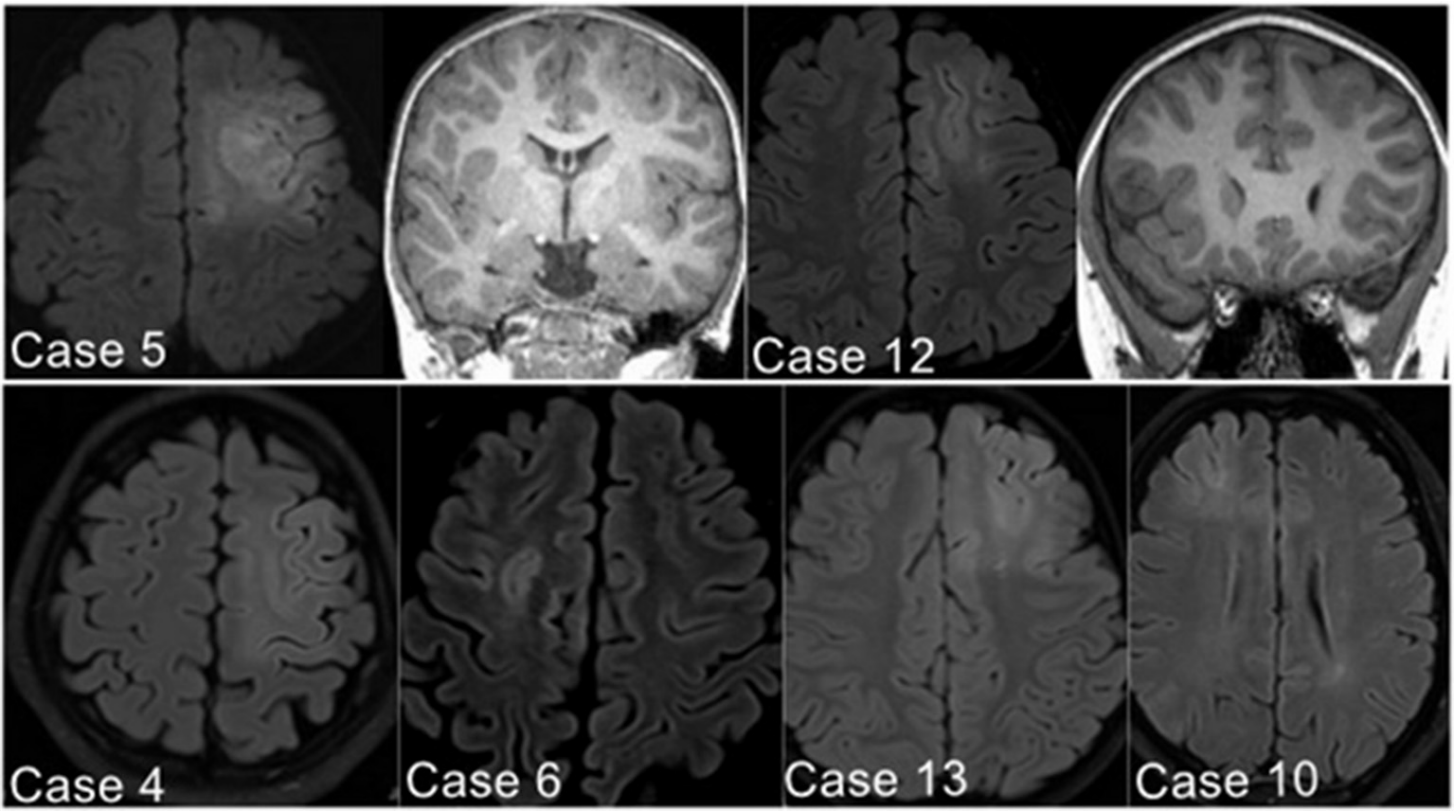
Illustrative MRI positive examples. Six patients in the PS group demonstrated the suspected FCD in the SFS, detected with axial image of Flair sequence (Case 10 located in the anterior part of left SFS; the other five cases found in the posterior part of SFS).

The presurgical MRI results of 11 patients detected focal abnormal signals (6/15 in the PS group, 2/5 in AS group, and 3/11 in the NS group). Six patients in the PS group demonstrated the suspected FCD in the SFS, which was located in the depth of the posterior part of the SFS in five patients (83.3%) and in the anterior part of the SFS in only one (Case 10 in Table 1) (Figure 2). Two patients in AS group also showed SFS-related abnormalities. The visible lesions shown in the MRI of the three patients of the NS group were all located in frontal lobe areas unrelated to the SFS. The other 20 patients (64.5%) had non-lesional MRI.

**Figure 3:**
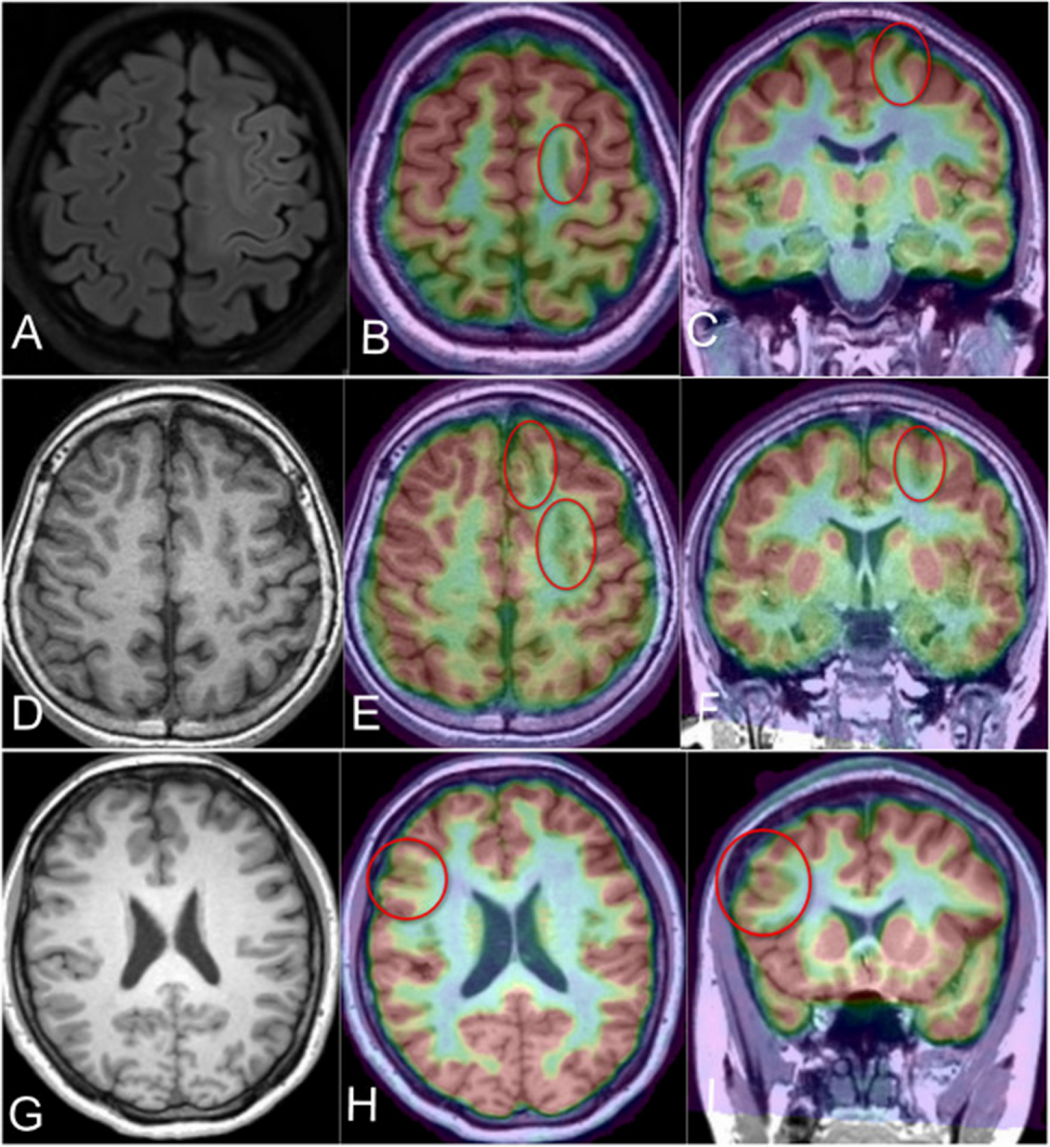
PET-MR co-registration in the MR-positive or MR-negative cases in different groups. **(A)** Case 4 in the PS group; the axial flair scan demonstrated the abnormal signal and gray-white matter blurring in the posterior part of left SFS (MR-positive), **(B, C)** the PET-MR co-registration image (axial and coronal) revealed the hypometabolism foci in the same location of MR image; **(D)** demonstrated the MR-negative Case 3 in the AS group, **(E, F)** showed the hypometabolism of the left SFS and adjacent sulcus (red circles); **(G)** demonstrated the T1-negative image of Case 2 in the NS group; **(H, I)** revealed the hypometabolism area in the right inferior frontal sulcus related lobule (red circle).

Interictal PET and 3D T1 were routinely co-registered for all 31 patients with the software SPM12. In this cohort, all patients demonstrated focal abnormal hypometabolism foci in the PET-MR co-registration, and the suspected lesion in MR-positive patients was consistent and confirmed with PET/MR (Figure 3).

### 3.3. Surgery and surgical outcome

All 31 frontal epilepsy patients underwent surgical resections. Fifteen PS group patients were considered the focal SFS-related EZ, who performed focal cortical resection in SFS. The surgical resections involved both the SFS-related lesion and adjacent frontal cortex in all five patients of AS group, and the patients of the NS group underwent the corticectomy which was unrelated to SFS.

Only transient postoperative complications were observed, including mild contralateral motor deficits for a few days in 7/15 patients of the PS group (46.7%), in 2/5 patients of AS group (40%), and 1/11 patients of the NS group (9.1%). The difference was significant in patients with (PS and AS groups) or without (NS group) SFS-related cortical resection (*p* = 0.021).

**Figure 4:**
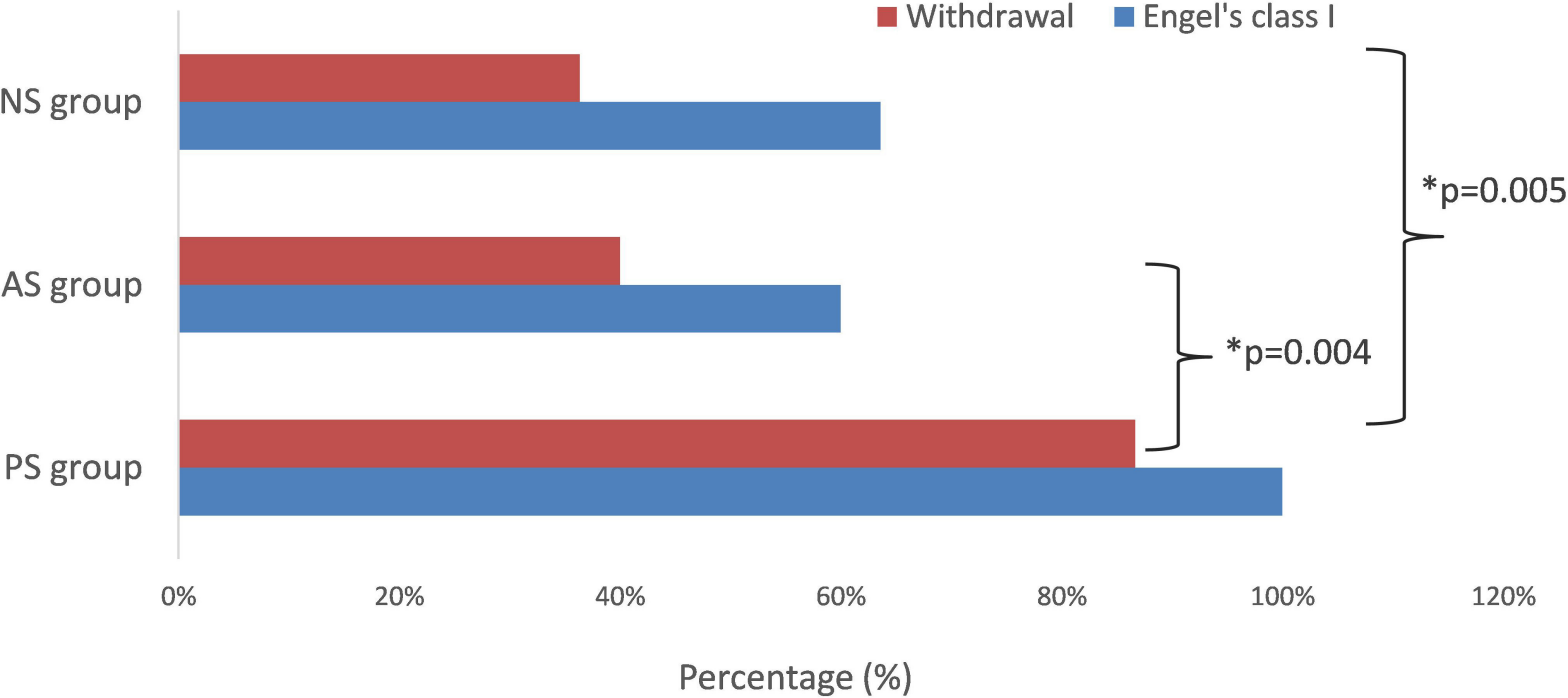
The seizure-free rate and withdrawal rate in different groups. The difference in seizure-free rate was significant (*p* = 0.004, PS vs. AS; *p* = 0.005, PS vs. NS). *means significant different.

Twenty-five patients (80.6%) were seizure-free since surgery (Engel's class I), including all 15 patients (100%) of the PS group, three in five patients (60%) of the AS group, and seven in 11 patients (63.6%) of the NS group. The difference in outcome between different groups was significant (*p* = 0.004, PS vs. AS; *p* = 0.005, PS vs. NS) (Figure 4). The mean follow-up time was 66.2 ± 9.7months (range 43–75 months). As of the last follow-up, antiseizure medication had been withdrawn in 19 patients (13 in the PS group, two in AS group, and four in the NS group) from seizure-free patients and had been tapered in six patients (two in the PS group, one in AS group, and three in NS group).

### 3.4. Histology

**Figure 5:**
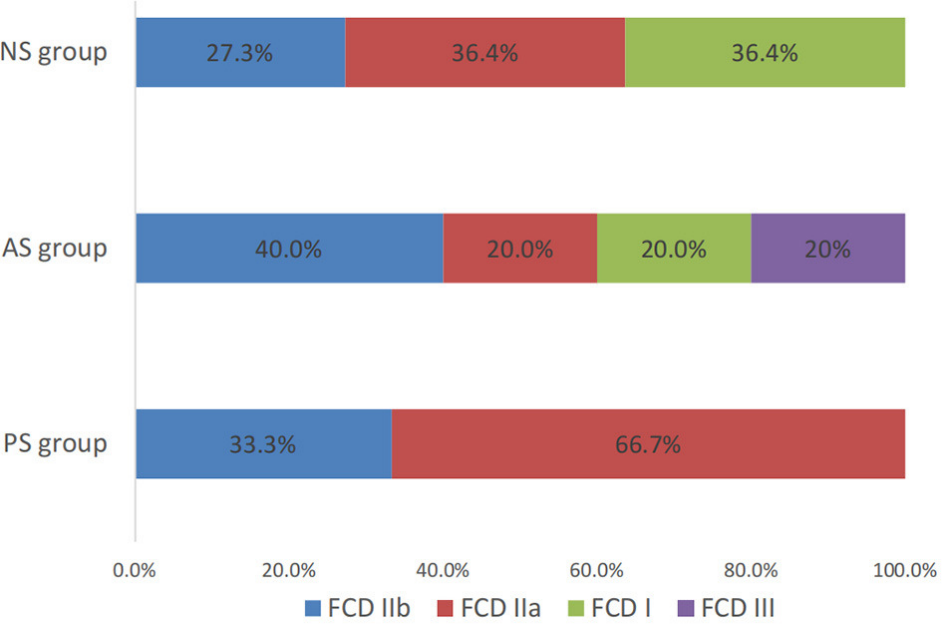
The pathological feature and the percentage of FCD type in different groups.

Histologic examination of resected specimens revealed FCD in all 31 patients. In the PS group, all 15 patients were FCD II type (five FCD IIb and 10 FCD IIa). There were three patients (60%) of FCD II type (two FCD IIb and one FCD IIa), one patient of suspected FCD IIId (with malacia after birth trauma) in AS group, and seven patients (63.6%) of FCD II type (three FCD IIb and four FCD IIa) in the NS group (Figure 5). Of the 25 patients with seizure-free surgical outcomes, their histopathologic evaluations showed FCD II in 23 patients.

## 4. Discussion

To the extent of our knowledge, our study presents the largest cohort undergoing SFS-related epilepsy surgery with the longest postoperative follow-up to date, enabling us to delineate the long-term effect and outcome on the rates of seizure freedom and drug withdrawal.

### 4.1. Frontal lobe epilepsy and superior frontal sulcus FCD

FLE is the common focal epilepsy secondary to temporal lobe epilepsy. The epileptic activity of the frontal lobe propagates rapidly, and localizing the epileptic network is complex even with SEEG (15). Our cohort has a male predominance (22 male, nine female) for FLE. The ratio of male to female patients is 2.4:1. The pathology of the 17 cases in 22 male patients is FCD II. Tassi et al. reported the electroclinical features of 100 FCD type II patients, there was no significant difference in gender between FCD IIa and FCD IIb (16). However, the authors did not address the difference in gender between FCD type II and other FCD type patients. Moreover, an earlier study reported by the same authors also had a similar result, which did not show significant differences between genders in relation to FCD type (17). It is recommended that gender differences in FCD type be focused on in future studies.

Leventer et al. reported a study of the types, frequencies, clinical characteristics, and MRI features of malformations of cortical development. However, the percentage of FCD was only 16%. The authors also found an association between MRI lesions located in the frontal lobe and FCD (18). Tassi et al. also reported that frontal lobe lesions are more likely to be associated with epilepsy (19). As we know, FLE mainly occurs during sleep, and the FCD IIb of FLE is the most frequent cause of sleep-related seizures (20). However, some studies have reported that type II FCD, regardless of its anatomical localization, increases the risk of sleep-related epilepsy (16, 21–23). In our cohort, people in the SFS-related groups (PS and AS groups) were less likely to have nocturnal seizures compared to the NS group, while the rate of type II FCD in SFS-related groups, especially in the PS group, is higher than that in the NS group. This is possibly why SFS-related type II dysplasia shows a specific pattern compared to other type II FCD, which is less likely to develop into a seizure during slow-wave sleep as other types. Therefore, the advanced exploration of the mechanism and molecular pathogenesis of SFS-related dysplasia, such as nocturnal FLE, is necessary.

FCD type II lesions are highly epileptogenic and often associated with medically intractable focal epilepsy (24, 25), especially FCD IIb. FCD IIb lesions are often located in the frontal lobe (10, 26). A similar study showed that the FCD was located mainly in the depth of the posterior part of the SFS and intermediate frontal sulcus (7, 8). According to our study, the presence of FCD in the depth of the posterior part of the SFS has a good surgical outcome.

### 4.2. Presurgical evaluation

A considerable number of epileptic lesions can be found by visual analysis of MRI, such as FCD IIb. Subtle FCDs are missed in up to 30% of patients in surgical cohorts (27). In our cohort, 20 patients (63.4%) were considered as MR-negative. Although considered MR-negative, the patients in our cohort demonstrated suspected hypometabolic FCD associated with or without SFS in the EZ according to the PET-MR co-registration images.

In the presurgical evaluation of refractory epilepsy, non-invasive localization is of substantial importance and can directly affect invasive evaluation and surgical resection. In addition, MRI post-processing, focusing on different features of subtle brain pathology, can serve as a valuable method to accurately identify cortical abnormalities. However, the relationship between the topography of the radiological lesion and the topography of the EZ in patients presenting with visible MRI or PET/MR lesions is complex. In these patients, some cases turn out to have the focal EZ centered in the lesion; similar to our PS group, the EZs are all centered in the SFS-related FCD. However, some cases have a more widespread EZ involving several cortical areas, with only one being adjacent to the radiological lesion, such as the AS group and NS group in our study. In addition, the cortex lesions in experimental models have been shown to induce distant remodeling of the cortex, leading to the onset of unusual patterns separated from the initial lesion (28). In our cohort, imaging abnormalities on MRI and/or PET tended to be well-circumscribed, which likely contributed to the good outcome after focal resections in this area.

### 4.3. Surgical outcome and SFS-related dysplasia

Our surgical results show that 25 patients (80.6%) with FLE had been seizure-free since surgery (Engel's class I) in long-term follow-up. In the PS group of 15 patients with SFS-related FCD on histopathology, all patients (100%) had seizure freedom and withdrawal of or tapered medication. This is significantly higher than the other two groups. Regis et al. and Nobili et al. also reported similar results, demonstrating a 73 and 81% seizure freedom rate among those with good correspondence between the EZ and the visible FCD (7, 29). However, Siegel et al. and Lorenzo et al. reported that the seizure freedom rate was lower than 57% (30, 31). The possible reason is that a successful outcome strongly correlates with having both focal frontal 3T MRI lesion and pathological abnormality, compared to the less favorable results in patients with a normal MRI. Based on this correlation between focal positive lesion and seizure freedom rate, FCD, which may be present in the EZ of the MR-negative FLE patients, can be precisely identified based on a comprehensive presurgical evaluation including PET and SEEG. It also addresses the lower rate of invasive EEG recordings in the PS group than the other two groups.

The surgical outcome is also correlated with the epilepsy duration at the time of surgery. Our study showed no significant difference in epilepsy duration between the three groups. This could be for the following reasons. Most patients in the PS (46.7%) and AS (60%) groups were considered surgical candidates at the time of the first 5 years with epilepsy (Figure 1). In addition, the seizure freedom rate is more likely to be high from the resection of the FCD type II. In our cohort, the rate of FCD type II in the PS group (100%) was higher than in the other two groups. SFS-related FCD, especially SFS-related FCD type II, is much more resistant to the medication and centered in lesion-related EZ than other pathological types.

Although there were no permanent neuro-deficits related to the surgery in our study, the transient complication was not uncommon. As mentioned above, the critical functional cortex is localized in the frontal lobe, such as the posterior SFS-related motor cortex, which is the possible reason for a higher complication rate in the PS group than in the other two groups. Therefore, the preoperative electrical mapping or intraoperative cortical electrical stimulation to define the precise motor cortex in the posterior SFS-related dysplasia resection surgery is substantially critical.

## 5. Conclusion

SFS-related FCD is a neuropathologic entity with a favorable postoperative outcome. It is favorable for early resection surgery. An incomplete resection of the cortical abnormality outside the SFS mainly causes surgical treatment failure in SFS-related dysplasia. FDG-PET co-registered with MRI should be performed in patients with suspected SFS-related dysplasia, since it may depict areas of hypometabolism suggestive of dysplasia in the absence of MRI abnormalities.

## Data availability statement

The original contributions presented in the study are included in the article/supplementary material, further inquiries can be directed to the corresponding authors.

## Ethics statement

The studies involving human participants were reviewed and approved by the Ethics Committee of Zhejiang Provincial People's Hospital. Written informed consent to participate in this study was provided by the participants' legal guardian/next of kin.

## Author contributions

YX: conceptualization and neurosurgeon to perform SEEG and writing original draft preparation. Y-SM and KZ: neurosurgeons to perform surgical resection. W-HH and X-QS: review clinical and EEG/SEEG data. LL and J-GZ: supervision, guide the design of the study, and writing—reviewing and editing. All authors contributed to the article and approved the submitted version.
